# Außergewöhnliche Berufsallergien durch Nahrungsmittel tierischen Ursprungs

**DOI:** 10.1007/s00105-021-04810-8

**Published:** 2021-04-20

**Authors:** H. Dickel

**Affiliations:** Klinik für Dermatologie, Venerologie und Allergologie, St. Josef-Hospital, Universitätsklinikum der Ruhr-Universität Bochum (UK RUB), Gudrunstr. 56, 44791 Bochum, Deutschland

**Keywords:** Fleisch, Kontakturtikaria, Meerestiere, Proteinkontaktdermatitis, Allergische Sofortreaktionen, Meat, Contact urticaria, Seafood, Protein contact dermatitis, Immediate allergic reactions

## Abstract

**Hintergrund:**

Die Nahrungsmittelwirtschaft zählt zu den Hochrisikobereichen für arbeitsbedingte allergische Sofortreaktionen an der Haut (Kontakturtikaria, Kontakturtikariasyndrom, Proteinkontaktdermatitis) mit oder ohne respiratorische Symptome (allergische Rhinitis, allergisches Asthma) durch Proteine tierischen Ursprungs.

**Fragestellung:**

Die vorliegende Arbeit gibt einen Überblick über allergene Meerestier- und Fleischproteine und deren klinische und beruflich Relevanz in verschiedenen Tätigkeitsbereichen.

**Material und Methode:**

Es erfolgten die Aufbereitung von aktuellem Wissen sowie eine ergänzende selektive Literaturrecherche.

**Ergebnisse:**

Nach dem irritativen Handekzem ist die Proteinkontaktdermatitis eine häufige Berufsdermatose unter Beschäftigten mit Kontakt zu Meerestieren und Fleisch. Fischer, Köche und Metzger sind an erster Stelle betroffen. Noch vor den Fischen zählen Krusten- und Weichtiere zu den häufigsten Auslösern von in Einzelfällen auch vital bedrohlichen Nahrungsmittelallergien. Demgegenüber ist eine primäre Fleischallergie selten. Rindfleisch unter den Säugetieren und Hühnerfleisch unter den Vögeln sind die häufigsten Auslöser.

**Schlussfolgerungen:**

Bei allen Beschäftigten mit allergischen Sofortreaktionen an der Haut und/oder den Atemwegen, die gegenüber Proteinen tierischen Ursprungs am Arbeitsplatz exponiert sind, sollte frühzeitig eine sorgfältige allergologische Abklärung durchgeführt werden. Spielt heute die spezifische Ig(Immunglobulin)E-Bestimmung bei der Diagnosesicherung eine zentrale Rolle, wird es in Einzelfällen von großer Bedeutung sein, das angeschuldigte Nativmaterial in die Hauttestungen einzubeziehen. Die Krankheitsverläufe sind allgemein durch eine schlechte Prognose und häufige Berufswechsel charakterisiert.

## Abstract (en)

**Background:**

The food industry is a high-risk area for work-related allergic immediate skin reactions (contact urticaria, contact urticaria syndrome, protein contact dermatitis) with or without respiratory symptoms (allergic rhinitis, allergic asthma) due to proteins of animal origin.

**Objectives:**

The present work gives an overview of allergenic seafood and meat proteins and their clinical and occupational relevance in different work settings.

**Methods:**

A review of current knowledge and a supplementary selective literature search were performed.

**Results:**

Protein contact dermatitis is one of the more common occupational dermatoses in workers exposed to seafood and meat, after irritant hand eczema. Fishermen, cooks, and butchers are most commonly affected. Crustaceans and mollusks are more frequent triggers of food allergies than fish and can in individual cases also be life threatening. In contrast, primary meat allergy is rare. Beef among mammals and chicken among birds are the most common triggers.

**Conclusions:**

All employees with allergic immediate skin and/or respiratory reactions exposed to proteins of animal origin in the workplace should undergo a careful allergological workup at an early stage. Determination of the specific IgE plays a central role in confirming the diagnosis and it is of great importance in individual cases to include the suspected native material in the skin tests. The course of the disease is generally characterized by a poor prognosis and is commonly associated with a change in occupation.

Arbeitsbedingte allergische Sofortreaktionen an der Haut mit oder ohne respiratorische Symptome durch Nahrungsmittel treten nicht selten bei Beschäftigten in bestimmten Risikoberufen auf, werden jedoch häufig nicht diagnostiziert. Der Erkrankungsverlauf ist zumeist prognostisch ungünstig, und früh ist eine Berufsaufgabe unausweichlich. In dem Beitrag werden Meerestier- und Fleischproteine mit ihrer allergologischen Relevanz in Risikoberufen behandelt. Auf die Haut und die Atemwege ausgerichtete arbeitsplatzbezogene Präventionsmaßnahmen stellen eine wichtige therapeutische Intervention dar.

Arbeitsbedingte Hauterkrankungen nehmen in vielen Industrieländern den ersten Platz unter den Berufskrankheiten ein [12], hierbei am häufigsten als irritatives und allergisches Kontaktekzem. Vergleichsweise seltener, wobei von einer Unterschätzung des Vorkommens auszugehen ist [69], sind allergische (Ig[Immunglobulin]E-vermittelte) Sofortreaktionen an der Haut zu beobachten [12], die allerdings das Risiko systemischer Reaktionen beinhalten [45].

Die allergischen Sofortreaktionen an der Haut umfassen eine heterogene Gruppe von Entzündungsreaktionen, die innerhalb von wenigen Minuten (ca. 15 min) nach Haut- oder Schleimhautkontakt mit dem auslösenden Allergen auftreten und in der Regel innerhalb weniger Stunden abklingen – wenngleich manchmal auch verzögert einsetzende Reaktionen (ca. 4–6 h) zu beobachten sind [41]. Zu den die allergischen Sofortreaktionen an der Haut kennzeichnenden Krankheitsentitäten zählen die immunologische (allergische) Kontakturtikaria, das Kontakturtikariasyndrom [71] und die Proteinkontaktdermatitis [19, 20, 52]. Symptome an den Schleimhäuten (orales Allergiesyndrom) bzw. eine begleitende Atemwegserkrankung (allergische Rhinitis und/oder allergisches Asthma), die durch das gleiche Allergen ausgelöst werden, sind nicht selten begleitend vorhanden.

Die allergischen Sofortreaktionen an der Haut können grob in durch Proteine (10 bis ≥ 10.000 kD) ausgelöste und in durch chemische Haptene (< 1 kD) ausgelöste unterteilt werden [20]. Die allergenen Proteine lassen sich grob in pflanzliche und tierische Proteine und Enzyme einteilen. In diesem Beitrag werden im Weiteren die Proteine tierischen Ursprungs – Meerestier- und Fleischproteine – mit ihrer allergologischen Relevanz in bestimmten Gruppen von Berufstätigen behandelt.

## Meerestiere

zählen zu den potentesten allergenen Nahrungsmitteln und somit zu den häufigsten Auslösern von Nahrungsmittelallergien [5, 66]. Zusätzlich gelten sie auch als wichtige Auslöser von arbeitsbedingten Allergien [10, 11]. Zu den Meerestieren werden Fische und Meeresfrüchte gezählt. Zu den beliebtesten Speisefischen hierzulande gehören Seelachs, Lachs, Thunfisch, Hering und Forelle [33, 37]. Die Meeresfrüchte umfassen Krusten- und Weichtiere [27]. Häufig verzehrte Krustentiere sind Garnele, Languste, Flusskrebs und Hummer [37], die beliebtesten Weichtiere Muscheln, Tintenfische und Kalmare [44].

## Fleisch

wird üblicherweise definiert als das Fleisch und die Innereien von Tieren (hauptsächlich Säugetiere) und Vögeln (hauptsächlich Geflügel) [54]. Die primäre Rindfleischallergie, das Katzen-Schweinefleisch-Syndrom (in einigen Fällen auch auf Rindfleisch ausgedehnt) und das α‑Gal (Galactose-α‑1,3‑Galactose)-Syndrom stellen die 3 derzeit anerkannten Formen der Allergie gegen Säugetierfleisch dar [76]. Generell ist eine Fleischallergie selten [54]. Rindfleisch unter den Säugetieren und Hühnerfleisch unter den Vögeln sind die häufigsten Auslöser.

## Epidemiologie

Das Auftreten von Meerestierallergien variiert stark in Abhängigkeit von länderspezifischen Essgewohnheiten, der Verbreitung an Meerestier-verarbeitender Industrie bzw. der Dichte an Gastronomie [37, 44]. Allgemein scheinen Allergien auf Meeresfrüchte häufiger als Fischallergien aufzutreten [8, 27]. Für die europäische Erwachsenenbevölkerung lassen sich auf der Grundlage von Selbsteinschätzung/Sensibilisierung/Anamnese + Sensibilisierung/Provokationstestung Prävalenzen von bis zu 1,5 % (95 %-KI [Konfidenzintervall] 1,0–2,2 %)/2,9 % (95 %-KI 2,2–3,9 %)/0,8 % (95 %-KI 0,2–2,5 %)/0,2 % (95 %-KI 0–0,9 %) für Fischallergien bzw. von bis zu 2,0 % (95 %-KI 1,2–3,3 %)/10,3 % (95 %-KI 7,0–14,9 %)/0,2 % (95 %-KI 0,1–0,5 %)/0,3 % (95 %-KI 0,1–1,0 %) für Meeresfrüchteallergien festhalten [49].

Allergien auf Meeresfrüchte scheinen häufiger als Fischallergien aufzutreten

Arbeitsbedingte Meerestierallergien sind in der Fischerei und Meerestier-verarbeitenden Industrie von großer Bedeutung. Wenngleich bevölkerungsbasierte Studien fehlen [64], wird die Prävalenz von immunologischer Kontakturtikaria bzw. Proteinkontaktdermatitis hier weltweit auf 3–11 % [28], die von allergischer Rhinitis auf 5–24 % [31, 43] und die von allergischem Asthma auf 2–36 % geschätzt [2, 17, 29, 31]. Das Berufsasthma wurde dabei häufiger mit Krustentieren (4–36 %) als mit Fischen (2–8 %) in Verbindung gebracht [29].

Auch bei Fleischallergien gibt es signifikante regionale Unterschiede, die auf Unterschiede in den lokalen Ernährungsgewohnheiten zurückzuführen sind. Aber auch andere Umweltfaktoren spielen eine Rolle, wie z. B. eine durch Zeckenbisse hervorgerufene IgE-vermittelte Sensibilisierung gegenüber α‑Gal [75]. Belastbare Schätzungen zur Prävalenz von Fleischallergien liegen nicht vor. In einer Studie mit 689 Erwachsenen in Pakistan fanden sich auf der Grundlage von Provokationstestungen Prävalenzen von 0,9 % (95 %-KI 0,4–1,9 %) für Rindfleischallergien und 1,0 % (95 %-KI 0,5–2,1 %) für Hühnerfleischallergien [25].

## Allergene

Die stetig steigende Anzahl auf molekularer Ebene charakterisierter allergener Meerestier- und Fleischproteine findet sich immer aktualisiert auf der offiziellen Website der Weltgesundheitsorganisation und der International Union of Immunological Societies für die systematische Nomenklatur für allergene Proteine aufgelistet (http://www.allergen.org; letzter Aufruf 15.03.2021) [35, 53, 75].

### Meerestierproteine

Bei Fischallergien sind in erster Linie Parvalbumine, Enolasen, Aldolasen und Kollagene als Allergene von Bedeutung [34, 48]. Bei Meeresfrüchteallergien wurden Tropomyosine, Argininkinasen, sarkoplasmatische kalziumbindende Proteine, Myosin-leichte-Ketten, Troponine, Triosephosphatisomerase und Hämocyanin als wichtige Allergene beschrieben [48, 55].

Unter den Meerestieren sind bislang zwischen Fischen und Meeresfrüchten keine Kreuzreaktionen bekannt [34]. Viele Fischallergiker reagieren jedoch auf eine ganze Bandbreite unterschiedlicher Fische [34]. Noch häufiger finden sich Kreuzreaktionen bei Meeresfrüchteallergikern, wobei Krustentiersensibilisierte nicht zwangsläufig auch auf Weichtiere sensibilisiert sind und umgekehrt [8, 55]. Bei Fischallergikern wurden darüber hinaus Kreuzreaktionen zu Hühnerfleisch (sog. „Fisch-Hühner-Syndrom“) und bei Meeresfrüchteallergikern zu Milben und Schaben (sog. „Milben-Meeresfrüchte-Syndrom“) beobachtet [23, 36, 75].

### Fleischproteine

Da Fleischallergien selten sind, sind molekulare Studien begrenzt, und nur wenige Proteine wurden bislang als Hauptallergene identifiziert [54]. Serumalbumin, das im Blutplasma der Tiere vorkommt, stellt einen der wichtigsten Auslöser sowohl bei Säugetier- als auch bei Vogelfleischallergien dar [75]. So ist Rinderserumalbumin eines der Hauptallergene bei Patienten mit Nahrungsmittelallergien, die durch Fleisch und Milch ausgelöst werden [53, 76]. Andere, weniger häufig identifizierte Allergene sind Immunglobulin, Myosin-leichte-Ketten-Kinase, Parvalbumin, Enolase und Aldolase [75, 76].

Aufgrund des hohen Grads an Homologie zwischen den Serumalbuminen ist die Wahrscheinlichkeit einer Kreuzreaktion hoch, wenn die Tiere phylogenetisch ähnlich sind [54, 76]. Rindfleischallergiker können beispielsweise auf Schweine- und Schaffleisch reagieren, nicht aber auf Vogelfleisch. In ähnlicher Weise können Allergiker, die auf Huhn reagieren, in der Regel keine Pute vertragen, vertragen aber Fleisch von Säugetieren. Bei Schweine- und seltener Rindfleischallergikern wurden darüber hinaus Kreuzreaktionen zum Serumalbumin der Katze (sog. „Katzen-Schweinefleisch-Syndrom“) und bei Pferdefleischallergikern zum Serumalbumin des Hundes (sog. „Hunde-Pferdefleisch-Syndrom“) beschrieben [75, 76].

Fleischallergien sind selten

Das Oligosaccharid (auch: Mehrfachzucker) α‑Gal ist selektiv nur auf Säugetiergewebe vorhanden [75]. Allergiker mit α‑Gal-Syndrom vertragen weiterhin Geflügelfleisch wie auch Meerestiere [77].

## Exponierte Arbeitsplätze

Arbeiter in der Tier‑, Forst- und Jagdwirtschaft, in der Nahrungsmittelherstellung, -verarbeitung, -zubereitung, -kontrolle und im Nahrungsmittelverkauf, in der Gastronomie und im Veterinärwesen zählen zu den Beschäftigten mit einem erhöhten Risiko für arbeitsbedingte allergische Sofortreaktionen durch Meerestiere und Fleisch [64]. Der Anteil an potenziell arbeitsbedingt Exponierten in diesen Berufsgruppen unter allen Beschäftigten darf hierzulande auf 6 % geschätzt werden [63].

Arbeitsbedingte Meerestierallergien sind bei (Tiefsee‑)Fischern, Fisch‑, Krabben- und Garnelenverarbeitern, Muschelöffnern, Austernschälern, Auslieferern von Meerestieren und Fischhändlern/-verkäufern beschrieben [28, 29, 31]. Im Bereich der Speisenzubereitung zählen Köche zu der herausragend exponierten Berufsgruppe [10, 11]. Die Sensibilisierung kann allgemein durch den ungeschützten Umgang mit Meerestieren und deren Produkten (z. B. Haut, Innereien, Saft) über die Haut sowie durch Einatmen von Aerosolen, die beim Hantieren, Schneiden, Schrubben oder Reinigen, Kochen oder Braten und Trocknen der Meerestiere entstehen, über die Atemwege erfolgen [28]. Zu den Arbeitsabläufen mit einem hohen Expositionspotenzial gegenüber Aerosolen gehört ferner das Reinigen der Prozesslinien oder der Lagertanks mit Wasserhochdruckreinigern.

Einem ständigen Kontakt am Arbeitsplatz zu allergieauslösenden Proteinen in verschiedenen Fleischsorten (Rind‑, Schweine‑, Geflügel‑, Lamm‑, Pferdefleisch), Innereien, Milch, Eiern usw. sind insbesondere Schlachthofarbeiter, Metzger und Wurstwarenhersteller, Köche, Küchenhelfer, Büfettkräfte, Käsehersteller, Barkeeper wie auch Tierärzte ausgesetzt [12, 18, 20, 24, 39–41, 46, 57, 67]. Metzger und Wurstwarenhersteller sowie Schlachthofarbeiter sind aufgrund ihres direkten Hautkontakts zu Tierdärmen besonders anfällig für die Entwicklung arbeitsbedingter Fleischallergien [40]. Neben Tierdärmen und Mesenterialfett sind Blut und Rinderfruchtwasser häufige Auslöser von arbeitsbedingten Fleischallergien bei Tierärzten, die beispielsweise in Schlachthöfen in der Fleischkontrolle tätig sind [40, 41]. Die Sensibilisierung wird bei der primären Rindfleischallergie mit der Exposition gegenüber dem relevanten Allergen über die Haut bzw. den Magen-Darm-Trakt angenommen [76]. In einer bevölkerungsbasierten Studie in Südwestdeutschland konnte unlängst nachgewiesen werden, dass Forstangestellte (Forstwirte, Holzfäller) und Jäger eine hohe Prävalenz an durch Zeckenbisse erworbener α‑Gal-sIgE-Positivität haben, einhergehend mit dem gehäuften Auftreten einer Säugetierfleisch-induzierten verzögerten Anaphylaxie – dem α‑Gal-Syndrom [13].

## Klinik

Arbeitsbedingt manifestiert sich an den Händen häufig initial ein irritatives (kumulativ-subtoxisches) Kontaktekzem, das durch hohe Feuchtbelastung bei gleichzeitigem Umgang mit primär hautreizenden Meerestier- und/oder Fleischbestandteilen (z. B. Säfte mit Enzymaktivität) sowie oft zusätzlich auf dem Boden einer atopischen Hautdiathese ausgelöst und unterhalten wird [6, 7, 9, 10, 21, 30, 69, 70]. Bei derart gestörter Hautbarriere kann sich dann im Sinne eines „Zweiphasenekzems“ eine immunologische Kontakturtikaria und mit zunehmender Chronizität eine Proteinkontaktdermatitis – Letztere als zweithäufigste Berufsdermatose bei Betroffenen mit arbeitsbedingtem Nahrungsmittelumgang [70] – aufpfropfen [56].

Arbeitsbedingt manifestiert sich an den Händen häufig initial ein irritatives Kontaktekzem

Als am Arbeitsplatz eine Kontakturtikaria bzw. Proteinkontaktdermatitis auslösende Nahrungsmittelallergene tierischen Ursprungs wurden Fisch (u. a. Flunder, Forelle, Hering, Kabeljau, Lachs, Makrele, Meerbarbe, Regenbogenforelle, Rotbarbe, Scampi, Schellfisch, Scholle, See- oder Meeraal, Seebrasse, Seeteufel, Seezunge, Stöcker, Thunfisch, Weißfisch, Wittling, Wolfsbarsch), Meeresfrüchte (u. a. Auster, Garnele, Hummer, Jakobsmuschel, Krabbe, Seeohr, Seespinne, Tintenfisch, Venusmuschel), Fleisch (u. a. von Huhn, Kalb, Lamm, Pferd, Pute, Reh, Rind, Schaf, Schwein), Milchprodukte (Parmesankäse) und Ei beschrieben [19, 39, 41, 52]. Die Proteinkontaktdermatitis kann sich nur als Ekzem der Fingerspitzen manifestieren oder sich auf Hände, Handgelenke und Unterarme ausdehnen [19, 68]. Bei aerogenem Hautkontakt durch z. B. freigesetzte Dämpfe können alle freigetragenen Körperstellen, wie beispielsweise Gesicht und Hals, mitbefallen sein [52, 64]. Das klinische Merkmal der Proteinkontaktdermatitis ist, dass akute Schübe von Juckreiz, Urtikaria, Ödemen oder Bläschenbildung wenige Minuten nach Kontakt mit dem allergenen Protein auftreten [40]. Einige Fälle von chronischer Paronychie werden als eine Variante der Proteinkontaktdermatitis betrachtet mit Rötung und Schwellung der proximalen Nagelfalze [19].

Insbesondere die Kontakturtikaria bzw. Proteinkontaktdermatitis auslösenden Meerestierallergene verursachen darüber hinaus nicht selten begleitende mukosale (orales Allergiesyndrom) und respiratorische Symptome (allergische Rhinitis und/oder allergisches Asthma) [19, 28, 40, 47, 53, 64]. Die allergischen Atemwegsbeschwerden treten im Allgemeinen nach einer Latenzzeit von Monaten bis Jahren auf [2, 10]. Als besonderer Fall findet sich ein arbeitsbedingtes neutrophiles fixes Nahrungsmittelexanthem bei einem Koch beschrieben, das sowohl durch Hautkontakt als auch nach Ingestion von Meerestieren ausgelöst wurde [73]. Meerestierallergene können außerdem lebensbedrohliche Anaphylaxien hervorrufen [14, 38, 42, 78], die sich meist nach Ingestion beobachten lassen [10, 14, 60]. Allerdings kann bei sehr hochgradiger Sensibilisierung schon der Hautkontakt alleine zu anaphylaktischen Reaktionen führen [10, 22, 28, 30].

Die primäre Fleischallergie manifestiert sich hauptsächlich als Sofortreaktion an der Haut nach Kontakt und weitaus seltener in Form von Übelkeit, Erbrechen und Anaphylaxie nach Ingestion [76]. Bei Vorliegen des Katzen-Schweinefleisch-Syndroms können Katzenallergiker systemische Reaktionen innerhalb der ersten Stunde nach Ingestion von Schweine- oder in Einzelfällen Rindfleisch zeigen [76]. Beim α‑Gal-Syndrom sind verzögerte anaphylaktische Reaktionen, typischerweise erst 3–6 h nach Ingestion von Säugetierfleisch, beschrieben [13, 76].

## Diagnostik

Die Diagnose einer arbeitsbedingten Meerestier- bzw. Fleischallergie beruht in der Regel auf Krankheits- und Expositionsanamnese, Allergietestungen sowie in Einzelfällen auf einer Provokationstestung [8, 12, 27, 55, 76]. Anamnesegeleitet werden spezifische IgE-Antikörper im Serum bestimmt und offene Anwendungstests (z. B. Reibtest) und/oder Pricktests durchgeführt [37, 65, 66, 76].

Allergenextrakte und -komponenten von verschiedenen Meerestieren sind zur spezifischen IgE-Bestimmung im Serum kommerziell verfügbar (Tab. 1 und 2; [37]). An Allergenkomponenten sind bereits die Fischparvalbumine von Karpfen und Kabeljau sowie das Garnelentropomyosin bestimmbar [32, 37, 78]. Zu den Allergenkomponententests, die bei der Beurteilung einer vermeintlichen (Säugetier‑)Fleischallergie besonders hilfreich sind, gehören neben den Allergenextrakttests Rinder‑, Schweine- und Katzenserumalbumin und α‑Gal (Tab. 3; [75, 76]).

**Table 1:** 

Nahrungsmittel – Fische	In-vitro-Testung	In-vivo-Testung			
	ImmunoCAP™ Allergene	Pricktestlösungen			
	Thermo Fisher Diagnostics^a^	ALK-Abelló Arzneimittel^b^	Bencard Allergie^c^	LETI Pharma^d^	ROXALL Medizin^e^
Aal	✓	–	–	–	–
Buntbarsch/Viktoriabarsch	✓	–	–	–	–
Forelle	✓	–	–	–	–
Golfflunder	✓	–	–	–	–
Heilbutt	✓	–	–	–	–
Hering	✓	✓	✓	–	–
Holzmakrele (Bastardmakrele)	✓	–	–	–	–
Kabeljau (Dorsch)	✓	–	✓	✓	✓
Kaiserbarsch	✓	–	–	–	–
Lachs	✓	–	–	–	–
Makrele	✓	–	–	–	–
Makrele, japanisch	✓	–	–	–	–
*Parvalbumin, Kabeljau (rGad c 1)*	✓	–	–	–	–
*Parvalbumin, Karpfen (rCyp c 1)*	✓	–	–	–	–
Plattfisch	✓	–	–	–	–
Roter Schnapper	✓	–	–	–	–
Sardelle	✓	–	–	–	–
Sardine	–	–	✓	–	–
Sardine (Mittelmeer)	✓	–	–	–	–
Sardine (Pazifik)	✓	–	–	–	–
Schellfisch	✓	–	–	–	–
Scholle	✓	–	✓	–	–
Schwertfisch	✓	–	–	–	–
Seehecht	✓	–	–	–	–
Seelachs	✓	–	–	–	–
Seezunge	✓	–	–	–	–
Thunfisch	✓	–	–	–	✓
Tintenfisch (Atlantik)	✓	–	–	–	–
Tintenfisch (Pazifik)	✓	–	–	–	–
Weißlachs	✓	–	–	–	–
Wels	✓	–	–	–	–
Zackenbarsch	✓	–	–	–	–
Zander, amerikanisch	✓	–	–	–	–

Aufgelistet sind nur die die entsprechenden Allergene anbietenden Firmen (ohne Gewähr für die Richtigkeit und Vollständigkeit der Angaben)

^a^Thermo Fisher Diagnostics GmbH, D‑79111 Freiburg

^b^ALK-Abelló Arzneimittel GmbH, D‑22763 Hamburg

^c^Bencard Allergie GmbH, D‑80804 München

^d^LETI Pharma GmbH, D‑58453 Witten

^e^ROXALL Medizin GmbH, D‑20535 Hamburg

**Table 2:** 

Nahrungsmittel – Meeresfrüchte	In-vitro-Testung	In-vivo-Testung		
	ImmunoCAP™ Allergene	Pricktestlösungen		
	Thermo Fisher Diagnostics^a^	ALK-Abelló Arzneimittel^b^	Bencard Allergie^c^	ROXALL Medizin^d^
Auster	✓	–	✓	–
Flusskrebs	✓	–	–	–
Garnele (Shrimps)	✓	–	–	✓
Hummer	✓	–	✓	–
Jakobsmuschel	✓	–	–	–
Krabbe	✓	✓	–	–
Languste	✓	–	–	–
Miesmuschel	✓	–	–	–
Oktopus	✓	–	–	–
Schnecke	✓	–	–	–
Seeohren (Abalone)	✓	–	–	–
*Tropomyosin, Garnele (rPen a 1)*	✓	–	–	–
Venusmuschel	✓	–	–	–

Aufgelistet sind nur die die entsprechenden Allergene anbietenden Firmen (ohne Gewähr für die Richtigkeit und Vollständigkeit der Angaben)

^a^Thermo Fisher Diagnostics GmbH, D‑79111 Freiburg

^b^ALK-Abelló Arzneimittel GmbH, D‑22763 Hamburg

^c^Bencard Allergie GmbH, D‑80804 München

^d^ROXALL Medizin GmbH, D‑20535 Hamburg

**Table 3:** 

Nahrungsmittel – Fleisch	In-vitro-Testung	In-vivo-Testung
	ImmunoCAP™ Allergene	Pricktestlösungen
	Thermo Fisher Diagnostics^a^	ROXALL Medizin^b^
Elchfleisch	✓	–
Hammelfleisch	✓	–
Hühnerfleisch	✓	✓
Kaninchenfleisch	✓	–
Lammfleisch	–	✓
Putenfleisch	–	✓
Rindfleisch	✓	✓
Schweinefleisch	✓	✓
*Serumalbumin, Rind (nBos d 6)*	✓	–
*Serumalbumin, Schwein (nSus s 1)*	✓	–
**Ergänzende In-vitro-Testung**		
*Alpha-Gal (nGal-alpha‑1,3‑Gal)*^e^	✓	–
Hühnereigelb^d^	✓	–
*Serumalbumin, Katze (rFel d 2)*^c^	✓	–

Aufgelistet sind nur die die entsprechenden Allergene anbietenden Firmen (ohne Gewähr für die Richtigkeit und Vollständigkeit der Angaben)

^a^Thermo Fisher Diagnostics GmbH, D‑79111 Freiburg

^b^ROXALL Medizin GmbH, D‑20535 Hamburg

^c^Hinweis auf α‑Gal-Syndrom

^d^Hinweis auf Vogel-Ei-Syndrom

^e^Hinweis auf Katzen-Schweinefleisch-Syndrom

Bei einem offenen Anwendungstest wird das Meerestier- bzw. Fleischmaterial auf die intakte Haut gelegt oder gerieben [18, 40, 50]. Da die Testergebnisse auf intakter Haut oft falsch negativ sind, ist es wichtig, das Nativmaterial auch auf läsionaler Haut zu testen. Der offene Anwendungstest gilt im Allgemeinen als weniger gefährlich als invasive Methoden wie der Pricktest. Daher empfehlen einige Autoren die offene Anwendungstestung vor der Durchführung invasiverer Tests [41].

Der offene Anwendungstest gilt als weniger gefährlich als invasive Methoden wie der Pricktest

Seit Allergietestlösungen aufgrund europäischer Rechtslage als Arzneimittel zugelassen werden müssen, ist die Auswahl an kommerziell erhältlichen Meerestier- bzw. Fleischallergenextrakten für den Pricktest immer stärker eingeschränkt worden (Tab. 1, 2 und 3; [4]). Zudem sind der Allergengehalt und die Standardisierung der Testlösungen häufig nicht zufriedenstellend [46]. Daher ist oftmals ein Prick-zu-Prick-Test mit frischem oder zubereitetem Nativmaterial erforderlich [12, 16, 24, 31, 46, 75, 78]. Der Prick-zu-Prick-Test – Einbettung der Hautpricklanzette in das Nativmaterial vor dem eigentlichen Prick [41] – kann gegenüber dem Pricktest mit kommerziell erhältlichen Allergenextrakten sensitiver sein [12, 51, 69, 77], v. a. wenn das Nativmaterial im gleichen Zubereitungsstadium vorliegt wie bei Auslösung der allergischen Reaktion [40, 69]. Beim Testen mit frischem Nativmaterial ist allgemein Vorsicht geboten im Hinblick auf eine mögliche Übertragung von Infektionskrankheiten; auf die Testung von Kontrollen ist zu verzichten [41].

Der Goldstandard für die Diagnostik von arbeitsbedingtem allergischem Asthma wird nur durch eine kontrollierte bronchiale Provokation mit subirritativen Konzentrationen des vermuteten Meerestier- bzw. Fleischallergens erreicht [2]. Der positive prädiktive Wert einer positiven unspezifischen Methacholin-Provokation zur Bestimmung des Vorliegens von Asthma beträgt dagegen nur etwa 10 %, wohingegen der negative prädiktive Wert etwa 99 % beträgt [2].

## Therapie und arbeitsplatzbezogene Prävention

Zum jetzigen Zeitpunkt gibt es keine Möglichkeit zur kausalen Therapie einer Meerestier- oder Fischallergie [27, 37, 55, 76]. Oftmals bleibt nur die strikte und dauerhafte Allergenkarenz [2, 12, 40, 77], d. h. die entsprechenden allergieauslösenden Proteine nicht nur am Arbeitsplatz, sondern auch im Privaten zu vermeiden. Topika wie kurzfristig angewandte, höher potente Glukokortikoide können bei ekzematösen Hautveränderungen die Entzündung verringern und den Heilungsprozess beschleunigen [40]. Bei Atemwegsbeschwerden wird therapeutisch die Kombination von entzündungshemmenden und bronchienerweiternden Medikamenten empfohlen [1].

Zum jetzigen Zeitpunkt gibt es keine kausale Therapie einer Meerestier- oder Fischallergie

Es ist wichtig, Beschäftigte mit arbeitsbedingten Hautbeschwerden auf Meerestiere bzw. Fleisch auch nach Atemwegsbeschwerden zu fragen und umgekehrt [47]. In positiven Fällen sollten wirksame Präventionsmaßnahmen den Schutz der Haut und der Atemwege gleichermaßen umfassen [10, 22]. Arbeitsplatz- und umgebungsbezogene wie auch diätetische Allergenkarenzstrategien sind umzusetzen [26, 31, 37, 76, 77]. Die präventiven Maßnahmen am Arbeitsplatz sind branchenabhängig und können z. B. Änderungen im Produktionsablauf, eine verbesserte Raumbelüftung, Absaugvorrichtungen oder Atemschutzgeräte umfassen [2].

Vor allem Meerestierallergiker leiden nicht zuletzt aufgrund der hohen Stabilität der Allergene lebenslang unter ihren Beschwerden [11, 15, 27, 55, 78], selbst nach Ende der arbeitsbedingten Exposition [28, 52]. Sie sind folglich über die möglichen Konsequenzen einer fortwährenden Allergenexposition bei ständig drohender Gefahr einer lebensbedrohlichen Anaphylaxie [58, 59] genau aufzuklären [42] und frühzeitig mit einem Notfallset – die Verschreibung von Epinephrin-Autoinjektoren beinhaltend – auszustatten [62, 74, 76, 77].

Es wurden bislang nur sehr wenige Daten zur relativen Stabilität verschiedener Fleischallergene veröffentlicht [54]. Fälle von allergischen Reaktionen auf rohes Fleisch bei gleichzeitiger Toleranz gegenüber gekochtem Fleisch wurden gefunden [54]. Verschiedene industrielle Fleischbehandlungsmethoden wie Erhitzen, Homogenisieren und Gefriertrocknen scheinen somit – anders als bei den Meerestieren [11] – das allergene Potenzial deutlich verringern zu können [54]. Häusliches Kochen ist dafür jedoch nicht ausreichend.

## Prognose

Im Vergleich zu einem irritativen oder allergischen Handekzem ist die Proteinkontaktdermatitis an den Händen bei Beschäftigten im nahrungsmittelverarbeitenden und -zubereitenden Bereich allgemein als prognostisch ungünstiger zu bewerten [3, 52]. Dies spiegelt sich unter anderem in häufigeren Arztkonsultationen, längeren Arbeitsunfähigkeitszeiten und häufigeren Berufswechseln wider [70].

Spätestens bei Hinzutreten systemischer bzw. anaphylaktischer Reaktionen im Rahmen des Kontakturtikariasyndroms [41, 71] wie auch davon losgelöst von oberen und/oder unteren Atemwegsbeschwerden [72] ist es kaum möglich, die Exposition gegenüber den teils potenten Nahrungsmittelallergenen tierischen Ursprungs am Arbeitsplatz sicher zu vermeiden. Eine berufliche Neuorientierung ist dann vielfach unausweichlich [11, 16, 31]. In einer eigenen retrospektiven Studie mit 30 Köchen mit einer arbeitsbedingten Meerestierallergie war eine Notfallbehandlung am Arbeitsplatz aufgrund eines anaphylaktischen Schocks in 17 % der Fälle notwendig geworden, und es resultierte letztlich in 90 % der Fälle die Berufsaufgabe im Median 6 Jahre nach Erkrankungsbeginn [10].

Mit Nachweis des klinisch und beruflich relevanten, am Arbeitsplatz nicht vermeidbaren Nahrungsmittelallergens tierischen Ursprungs bleibt das Vorliegen einer Berufskrankheit nach Nr. 5101 und/oder Nr. 4301 der Anlage zur Berufskrankheitenverordnung zu prüfen [11, 61].

## Fazit für die Praxis

- Vor allem Beschäftigte in der Nahrungsmittelherstellung, -verarbeitung, -zubereitung, -kontrolle und im Verkauf haben ein erhöhtes Risiko für arbeitsbedingte allergische Sofortreaktionen an der Haut (Kontakturtikaria, Kontakturtikariasyndrom, Proteinkontaktdermatitis) durch Meerestiere und Fleisch.
- Für Dermatologen ist es wichtig, die Proteinkontaktdermatitis in der Differenzialdiagnose des chronischen Handekzems stets mit zu berücksichtigen.
- Eine begleitende allergische Atemwegserkrankung, verursacht durch die gleichen, v. a. Meerestierallergene, tritt nicht selten auf und sollte mit abgeklärt werden.
- Um fallbezogen die diagnostische Lücke zu schließen, ist es erforderlich, neben spezifischer Ig(Immunglobulin)E-Bestimmung und Pricktestung mit kommerziellen Allergenextrakten offene Anwendungstests und/oder Prick-zu-Prick-Tests mit dem angeschuldigten Nativmaterial durchzuführen.
- Arbeitsplatzbezogene Präventionsmaßnahmen müssen den Schutz der Haut und der Atemwege gleichermaßen umfassen.
